# Trends in antimicrobial resistance of *Shigella* species in Peru, 2011–2020

**DOI:** 10.1093/jacamr/dlad110

**Published:** 2023-10-26

**Authors:** Willi Quino, Gustavo Bellido, Diana Flores-León, Junior Caro-Castro, Orson Mestanza, Jorge Lucero, Ronnie G Gavilan

**Affiliations:** Laboratorio de Referencia Nacional de Bacteriología Clínica, Instituto Nacional de Salud, Lima, Perú; Laboratorio de Referencia Nacional de Bacteriología Clínica, Instituto Nacional de Salud, Lima, Perú; Laboratorio de Referencia Nacional de Bacteriología Clínica, Instituto Nacional de Salud, Lima, Perú; Escuela Profesional de Medicina Humana, Universidad Privada San Juan Bautista, Lima, Perú; Laboratorio de Referencia Nacional de Bacteriología Clínica, Instituto Nacional de Salud, Lima, Perú; Laboratorio de Referencia Nacional de Bacteriología Clínica, Instituto Nacional de Salud, Lima, Perú; Laboratorio de Referencia Nacional de Bacteriología Clínica, Instituto Nacional de Salud, Lima, Perú; Laboratorio de Referencia Nacional de Bacteriología Clínica, Instituto Nacional de Salud, Lima, Perú; Escuela Profesional de Medicina Humana, Universidad Privada San Juan Bautista, Lima, Perú

## Abstract

**Objective:**

To describe the frequency of antimicrobial resistance rates and spatial-temporal distribution of *Shigella* species from the last 10 years in Peru.

**Methods:**

A cross-sectional descriptive study was carried out. A total of 1668 *Shigella* strains, remitted as part of the national enteric pathogen surveillance from 2011 to 2020, were analysed. The strains were confirmed by conventional tests and serotyped with polyvalent and monovalent antibodies. Also, antimicrobial susceptibility was performed according to the Kirby–Bauer method.

**Results:**

The most frequent *Shigella* species was *S. sonnei* (49.2%), followed by *S. flexneri* (42.2%), *S. boydii* (7.9%) and *S. dysenteriae* (0.7%). Phase II (46.29%) was the most frequent serotype in *S. sonnei*, serotype 2a (43.61%) in *S. flexneri*, serotype 2 in *S. boydii* and serotype 4 in *S. dysenteriae*. High rates of resistance were detected for trimethoprim/sulfamethoxazole (91.0%), tetracycline (88.4%), ampicillin (73.9%) and chloramphenicol (64.9%), moderate rates for amoxicillin/clavulanic acid (25.1%), ciprofloxacin (16.7%) and nalidixic acid (14.8%), and low rates for cefotaxime (1.74%), nitrofurantoin (0.7%) and ceftazidime (0.6%). Moreover, antimicrobial resistance to fluoroquinolones increased considerably from 2017 to 2020.

**Conclusion:**

*S. sonnei* was the most frequent species, which have a large proportion of strains resistant to trimethoprim/sulfamethoxazole, and a growing trend of resistance to ciprofloxacin and nalidixic acid. This increase in resistance to commonly used antibiotics in treatments is alarming, threatening the control and management of these currently treatable infections.

## Background

Diarrhoea is estimated to account for more than 1 million deaths and about 4% of the world’s total disability-adjusted life years per year in all age groups.^1–3^ Shigellosis represents the second leading cause of death from diarrhoea in the world (212 400 deaths per year),^1,4,5^ and one of the three leading causes of death from diarrhoea in children under 5 years of age.^4,6^ Shigellosis is an acute intestinal infection caused by the genus *Shigella*. It is divided into four serogroups with 47 serotypes: A (*S. dysenteriae*, 12 serotypes); B (*S. flexneri*, 15 serotypes), C (*S. boydii*, 18 serotypes) and D (*S. sonnei*, two antigenic types, phase I and phase II),^7,8^ whose transmission is by the faecal-oral route, with an infective dose of 10–100 bacteria. Countries with a low socioeconomic level are mainly affected by this disease.^5,9,10^

Antibiotic therapy is indicated in patients with moderate or severe symptoms to reduce the duration and severity of the disease, the transmission and shedding of organisms and to prevent lethal complications.^5,9,10^ However, in recent decades, different species and serotypes of *Shigella* that are resistant to traditional first-line antibiotics have emerged in several countries such as Peru, representing a serious public health problem in the world.^5,9–11^ Faced with this problem, the WHO recommended the use of ciprofloxacin as the first-line antibiotic for the treatment of all cases with bloody diarrhoea, and ceftriaxone and azithromycin as second-line antibiotics.^10^

Currently, *Shigella* strains resistance to ciprofloxacin has been increasing.^5,11^ Resistance to alternative drugs, such as ceftriaxone and azithromycin, has also been reported.^12,13^ If this trend continues, the management and control of shigellosis can be seriously affected. In addition to this problem, the scarcity of resources, the high rate of infectious diseases and the free access to antimicrobials would further complicate the management of shigellosis, reducing treatment options.^5,12,14^ In that sense, the WHO has included resistance to fluoroquinolones among the serious threats of antimicrobial resistance (AMR), for which strict surveillance is needed, as well as promoting urgent research and development of new antibiotics.^15–17^

In Perú, *Shigella* continues to be one of the main etiological agents of bacillary dysentery, which especially affects children and the elderly people.^18–22^ The increase of MDR (resistance to more than two classes of antimicrobials) threatens the prevention and control of shigellosis in our country.^5,9,23,24^ In this context, the aim of this study was to describe the frequency of AMR rates and spatial-temporal distribution of *Shigella* species from the last 10 years in Peru.

## Methods

### Ethics approval

This research was conducted in accordance with the Declaration of Helsinki and within the framework of the national AMR surveillance approved by the Instituto Nacional de Salud of Peru and the Committee of Research and Ethics. This approval was waived in accordance with the national legislation and the institutional requirements for Public Health Surveillance (S.D. no. 010-2019-SA).

### Study design and population

This is a retrospective and cross-sectional study. *Shigella* is routinely investigated in public and private health laboratories in Peru, but the notification in Peru is not mandatory. The strains are shipped to the Instituto Nacional de Salud when the other laboratories need confirmation of species, serotype and/or antimicrobial susceptibility. Therefore, the present study constitutes a passive report.

The population consisted of all *Shigella* strains remitted to the Instituto Nacional de Salud of Peru from 14 of the 25 regions of Peru, and recovered from 2011 to 2020. These strains were received by the National Reference Laboratory of Clinical Bacteriology under the framework of National Enteric Pathogens Surveillance.

To evaluate the distribution of *Shigella* strains over the time covered by the study, the date of collection of each strain were loaded into the R software v.4.3.1 to generate a violin plot using the ggplot2 library.

### Culture and typing of *Shigella* species and serotypes

All *Shigella* strains were previously cultured in trypticase soy broth (Oxoid, England) for 6 to 8 hours; Subsequently, each strain was plotted onto *Salmonella*–*Shigella* agar (Oxoid, England) and incubated at 37°C from 18 to 24 h. Presumptive identification was carried out using biochemical tests consisting of triple sugar iron agar, lysine iron agar, mobility indole ornithine agar and Simmons citrate agar (Oxoid, England). The species and serotype were determined using commercially available polyvalent and monovalent antisera (Denka Seiken Co., Ltd, Japan).

### Antimicrobial susceptibility test

Antimicrobial susceptibility was performed on Mueller–Hinton Agar using the Kirby–Bauer method.^25^ The total *Shigella* strains were analysed according to the interpretation criteria proposed by the CLSI.^26^ The antibiotics used were: ampicillin (10 μg), amoxicillin/clavulanate (20/10 μg), cefotaxime (30 μg), ceftazidime (30 μg), chloramphenicol (30 μg), ciprofloxacin (5 μg), tetracycline (30 μg), trimethoprim/sulfamethoxazole (1.25/23.75 μg), nalidixic acid (30 μg) and nitrofurantoin (300 μg) (Oxoid, England). For quality control, the reference strains *Escherichia coli* ATCC 25922, *Klebsiella pneumoniae* ATCC 700603 and *Pseudomonas aeruginosa* ATCC 27853 were used. The strains were considered MDR when they presented resistance to more than two classes of antimicrobials.^25^ Additionally, the determination of the production of ESBL was carried out using the Jarlier method.^27^ The presence of ESBL was manifested by the synergistic effect between the inhibitor (amoxicillin/clavulanate) and the cephalosporin antimicrobial discs (cefotaxime and ceftazidime).

## Statistical methods

Descriptive statistics were used to analyse associations between the gender of the patients and their age or the *Shigella* species isolated, as well as AMR rates between the most frequent *Shigella* species. The differentiations between groups were analysed by Pearson’s chi-square and a 95% confidence interval using IBM SPSS Statistic 23.

## Results

### Epidemiological analysis

**Figure 1.**
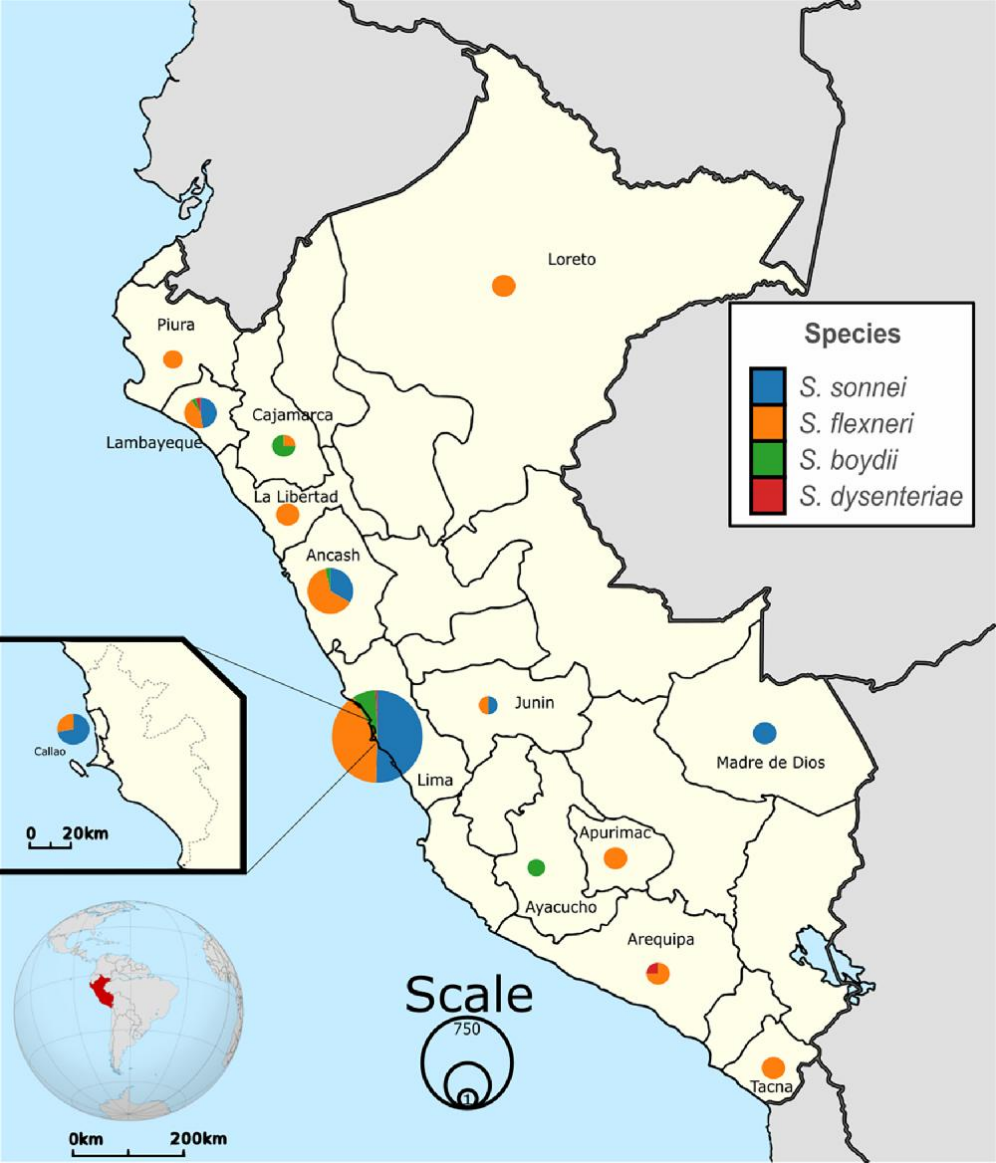
Map of Peru indicating the geographical distribution where *Shigella* strains were isolated. The size of the nodes indicates the number of species recovered in each region (according to the scale), while colors indicates *Shigella* species according to the legend. At least, one clinical strain from 14 Peruvian regions was recovered for this study.

At least, one *Shigella* strain from 14 Peruvian regions were included in this study: Ancash (*n* = 90), Apurímac (*n* = 3), Arequipa (*n* = 4), Ayacucho (*n* =1), Cajamarca (*n* = 4), Callao (*n* = 25), Junin (*n* = 2), La Libertad (*n* = 3), Lambayeque (*n* = 21), Lima (*n* = 1506), Loreto (*n* = 3), Madre de Dios (*n* = 2), Piura (*n* = 1) and Tacna (*n* = 3). Lima was the region with the highest frequency of *Shigella* strains recovered from 2011 to 2020 (90.29%) (Figure 1).

Of the total *Shigella* strains included in this study (*n* = 1668), 46.46% were isolated from male patients, 45.02% from females and 8.52% did not record data. *Shigella* isolation decreased with increasing age. From all species, the highest rate of isolation was observed in children from 0 to 5 years (67.33%), following a similar tendency by species, especially in *S. sonnei*, *S. flexneri* and *S. boydii*. An additional file shows this data in more detail (Table S1, available as Supplementary data at *JAC-AMR* Online). Moreover, no association was found between the gender of the patients and the age or the *Shigella* species isolated (*P* > 0.05) (Table 1).

**Table 1.** Chi-square test results on the relationship of age and *Shigella* species with the gender of patients

Characteristic	Gender		*χ* ^2^	*P* value*
	Male	Female		
Age				
0–5	554	517	3.29	0.19
6–18	136	146		
19–≤60	28	17		
Species				
*S. sonnei*	383	377	0.74	0.69
*S. flexneri*	305	329		
*S. boydii*	59	62		

* *P* < 0.05: Association between variables.

### Frequency of *Shigella* species and serotypes

**Figure 2.**
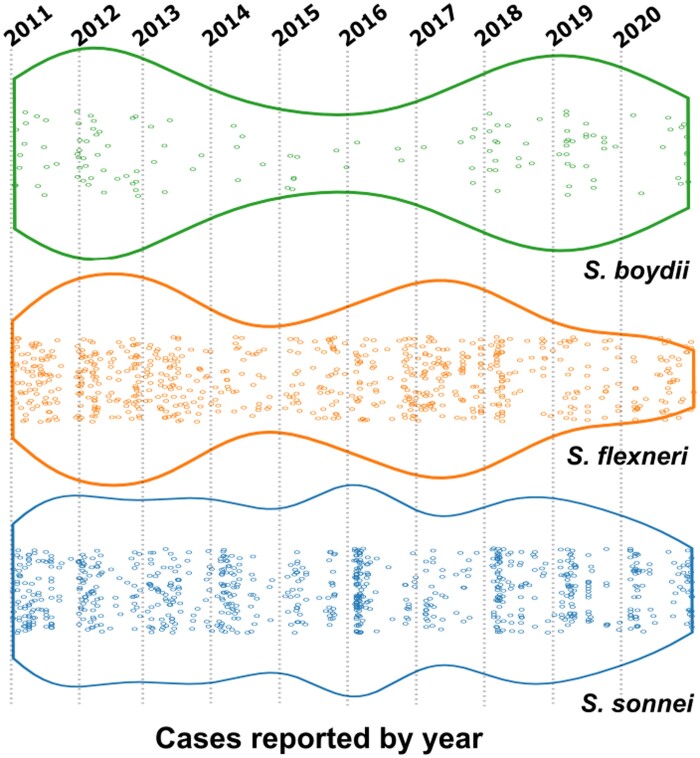
Temporal distribution of *Shigella* species by year of isolation in Peru. *S. flexneri* and *S. sonnei* have been the most frequent species throughout the study period, followed by *S. boydii*. The recovery rate of *S. sonnei* was constant, in contrast to the other three species that were heterogeneous.

The predominated Shigella species in this study was *S. sonnei* with 49.2% (*n* = 821), followed by *S. flexneri* with 42.2% (*n* = 704), *S. boydii* with 7.9% (*n* = 131) and *S. dysenteriae* with 0.7% (*n* = 12). According to the temporal analysis, the recovery rate of *S. sonnei* was constant, with little variations from 2011 to 2020, while in the other three species, their recovery rate was heterogeneous in the same period of time (Figure 2). Also, no strain of *S. dysenteriae* was recorded in the years 2015, 2016, 2019 and 2020. In fact, *S. dysenteriae* was the species with the lowest frequency in this study.

On the other hand, the most frequent serotypes in *S. sonnei* were phase II (46.29%) and phase I (41.05%), while in *S. flexneri* was serotype 2a (43.61%), in *S. boydii* was serotype 2 (51.91%) and in *S. dysenteriae* was serotype 4 (33.33%) (Table 2).

**Table 2.** Distribution of *Shigella* serotypes by species from 2011 to 2020

Species	Serotype	2011	2012	2013	2014	2015	2016	2017	2018	2019	2020	Total
		*N* = 193	*N* = 238	*N* = 175	*N* = 123	*N* = 105	*N* = 216	*N* = 147	*N* = 177	*N* = 140	*N* = 154	*N* = 1668
		*n* (%)	*n* (%)	*n* (%)	*n* (%)	*n* (%)	*n* (%)	*n* (%)	*n* (%)	*n* (%)	*n* (%)	*n* (%)
*Shigella sonnei*	NT	96 (49.7)	3 (1.3)	0	1 (0.8)	0	4 (1.9)	0	0	0	0	104 (6.2)
	1	0	59 (24.8)	50 (28.6)	42 (34.1)	29 (27.6)	34 (15.7)	5 (3.4)	54 (30.3)	26 (18.6)	38 (24.7)	337 (20.2)
	2	0	23 (9.7)	31 (17.7)	37 (30.1)	27 (25.7)	86 (39.8)	35 (23.8)	42 (23.6)	54 (38.6)	45 (29.2)	380 (22.8)
*Shigella flexneri*	NT	18 (9.3)	1 (0.4)	3 (1.7)	1 (0.8)	0	2 (0.9)	0	0	2 (1.4)	0	27 (1.6)
	1	0	1 (0.4)	0	0	0	0	0	0	1 (0.7)	0	2 (0.1)
	1a	2 (1.0)	7 (2.9)	3 (1.7)	2 (1.6)	0	2 (0.9)	5 (3.4)	9 (5.1)	2 (1.4)	0	32 (1.9)
	1b	6 (3.1)	22 (9.2)	15 (8.6)	11 (8.9)	10 (9.5)	19 (8.8)	29 (19.7)	15 (8.4)	9 (6.4)	5 (3.2)	141 (8.5)
	2	0	0	0	0	0	0	0	0	2 (1.4)	0	2 (0.1)
	2a	36 (18.7)	58 (24.4)	37 (21.1)	15 (12.2)	15 (14.3)	49 (22.7)	39 (26.5)	23 (12.9)	12 (8.6)	23 (14.9)	307 (18.4)
	2b	0	0	2 (1.1)	0	0	0	3 (2.0)	0	2 (1.4)	4 (2.6)	11 (0.7)
	3a	14 (7.3)	10 (4.2)	12 (6.9)	3 (2.4)	2 (1.9)	1 (0.5)	3 (2.0)	3 (1.7)	0	6 (3.9)	54 (3.2)
	3b	2 (1.0)	0	0	1(0.8)	1 (1.0)	0	1 (0.7)	3 (1.7)	0	0	8 (0.5)
	4	1 (0.5)	6 (2.5)	7 (4.0)	2 (1.6)	9 (8.6)	8 (3.7)	11 (7.5)	9 (5.1)	4 (2.9)	10 (6.5)	67 (4.0)
	4a	1 (0.5)	2 (0.8)	1(0.6)	1 (0.8)	0	0	1 (0.7)	0	0	0	6 (0.4)
	4b	0	0	0	0	0	0	0	1 (0.6)	0	2 (1.3)	3 (0.2)
	5a	1 (0.5)	0	0	0	0	0	0	0	0	0	1 (0.1)
	6	0	6 (2.5)	3 (1.7)	1 (0.8)	1 (1.0)	5 (2.3)	4 (2.7)	0	2 (1.4)	6 (3.9)	28 (1.7)
	x	0	0	1 (0.6)	0	0	1 (0.5)	2 (1.4)	1 (0.6)	0	1 (0.6)	6 (0.4)
	y	0	2 (0.8)	2 (1.1)	0	4 (3.8)	0	0	0	0	0	9 (0.5)
*Shigella boydii*	NT	2 (1.0)	2 (0.8)	0	3(2.4)	2 (1.9)	0	0	0	2 (1.4)	0	11 (0.7)
	2	7 (3.6)	0	0	1 (0.8)	3 (2.9)	5 (2.3)	5 (3.4)	16 (9.0)	19 (13.6)	12 (7.8)	68 (4.1)
	3	0	0	0	0	0	0		0	1 (0.7)	0	1 (0.1)
	4	4 (2.1)	5 (2.1)	1 (0.6)	0	0	0	1 (0.7)	0	0	1(0.6)	12 (0.7)
	10	1 (0.5)	4 (1.7)	1 (0.6)	0	0	0	0	0	2 (1.4)	1 (0.6)	9 (0.5)
	14	0	2 (0.8)	0	0	0	0	0	0	0	0	2 (0.1)
	18	0	0	0	1 (0.8)	0	0	0	0	0	0	1 (0.1)
	C	0	21 (8.8)	5 (2.9)	0	1 (1.0)	0	0	0	0	0	27 (1.6)
*Shigella dysenteriae*	NT	0	3 (1.3)	1 (0.6)	0	0	0	0	1 (0.6)	0	0	5 (0.3)
	2	1 (0.5)	1 (0.4)	0	1 (0.8)	0	0	1 (0.7)	0	0	0	4 (0.2)
	3	1 (0.5)	0	0	0	0	0	0	0	0	0	1 (0.1)
	7	0	0	0	0	0	0	2 (1.4)	0	0	0	2 (0.1)

NT, Nontypeable; C, Polivalent C (1-7).

### Antimicrobial susceptibility

From all *Shigella* strains (*n* = 1668), only 2 (0.12%) were susceptible to the 10 tested antimicrobials. Resistance to trimethoprim/sulfamethoxazole was the most frequent (91.0%), followed by tetracycline (88.4%), ampicillin (73.9%), chloramphenicol (64.9%), amoxicillin/clavulanic acid (25.1%), ciprofloxacin (16.7%), nalidixic acid (14.8%), cefotaxime (1.74%), nitrofurantoin (0.7%) and ceftazidime (0.6%).

Antibiotic resistance rates were different between *S. sonnei* and *S. flexneri.* The biggest resistance rates of *S. flexneri* were detected in tetracycline (84.4%), trimethoprim/sulfamethoxazole (84.1%) and ampicillin (74.6%). Similarly, the rate of resistance to trimethoprim/sulfamethoxazole (96.6%) and tetracycline (93.4%) were the highest in *S. sonnei*, followed by ampicillin (75.3%) and chloramphenicol (72.4%). Also, both *S. sonnei* and *S. flexneri,* showed little resistance to nitrofurantoin, ceftazidime and cefotaxime. Moreover, *S. flexneri* resistance to amoxicillin/clavulanic acid was slightly higher than in *S. sonnei* (Table 3).

**Table 3.** Antibiotic resistance in *Shigella* strains by subspecies

Antibiotic	*S. sonnei*	*S. flexneri*	*S. boydii*	Total^a^ *N* = 1668	Chi-square *P* value^b^
	*N* = 821	*N* = 704	*N* = 131		
	*n* (%)	*n* (%)	*n* (%)	*n* (%)	
SXT	793 (96.6)	592 (84.1)	122 (93.1)	1518 (91.0)	<0.001^c^
TET	767 (93.4)	594 (84.4)	110 (84.0)	1475 (88.4)	<0.001^c^
AMP	618 (75.3)	525 (74.6)	85 (64.8)	1234 (74.0)	0.75
CHL	594 (72.4)	476 (67.6)	10 (7.6)	1082 (64.9)	0.04^c^
CIP	235 (28.6)	32 (4.6)	11 (8.4)	279 (16.7)	<0.001^c^
NA	212 (25.8)	22 (3.1)	12 (9.2)	246 (14.8)	<0.001^c^
AMC	210 (25.6)	194 (27.6)	14 (10.7)	419 (25.1)	0.38
NIT	6 (0.7)	5 (0.7)	1 (0.8)	12 (0.7)	0.96
CAZ	5 (0.6)	4 (0.6)	1 (0.8)	10 (0.60)s	0.92
CTX	17 (2.1)	11 (1.6)	1(0.8)	29 (1.7)	0.46

SXT, trimethoprim/sulfamethoxazole; TET, tetracycline; AMP, ampicillin; CHL, chloramphenicol; CIP, ciprofloxacin; NA, nalidixic acid; AMC, Amoxicillin/clavulanic acid; NIT, nitrofurantoin; CAZ, ceftazidime; CTX, cefotaxime.

^a^The total includes *S. dysenteriae*.

^b^Comparison between *S. sonnei* and *S. flexneri*.

^c^ *P* < 0.05.

Resistance rates in *S. boydii* to trimethoprim/sulfamethoxazole, tetracycline and ampicillin were 93.1%, 84.0% and 64.8%, respectively. Trimethoprim/sulfamethoxazole resistance rates in *S. boydii* and *S. dysenteriae* were higher than in *S. flexneri*. In addition, a statistically significant association was found between AMR to trimethoprim/sulfamethoxazole, tetracycline, chloramphenicol, ciprofloxacin and nalidixic acid in S. *sonnei* and *S. flexneri* (*P* < 0.05) (Table 3).

**Figure 3.**
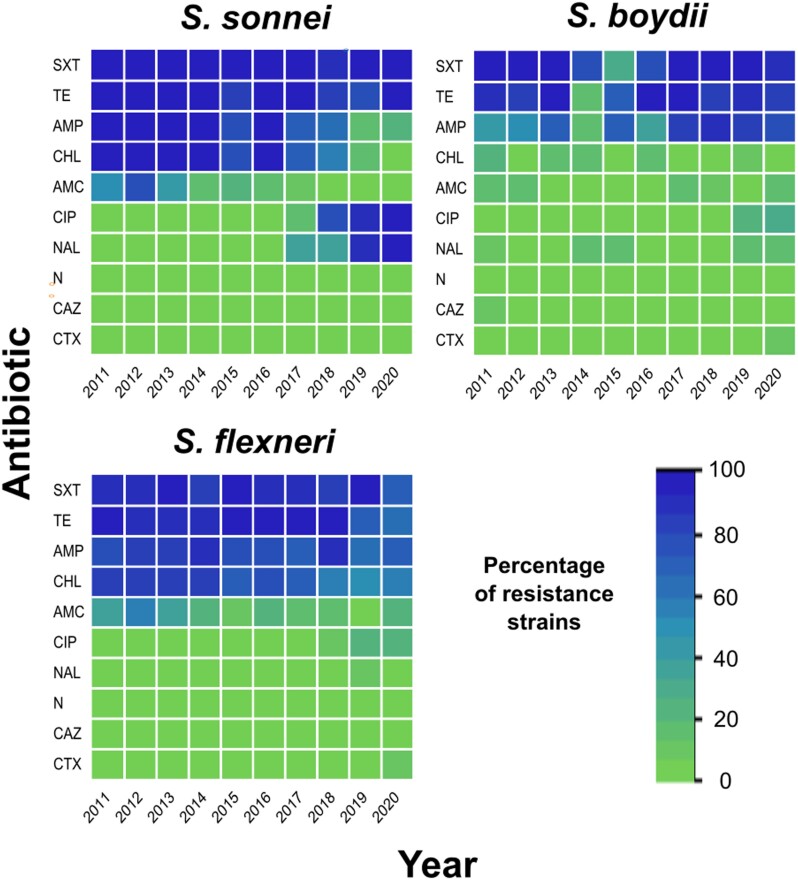
Heat maps of antibiotic resistance in *Shigella* strains by species from 2011 to 2020. SXT, trimethoprim/sulfamethoxazole; TET, tetracycline; AMP, ampicillin; CHL, chloramphenicol; CIP, ciprofloxacin; NA, nalidixic acid; AMC, amoxicillin/clavulanic acid; NIT, nitrofurantoin; CAZ, ceftazidime; CTX, cefotaxime.

Moreover, notable differences in antibiotic resistance profiles were also observed, especially those that changed significantly from 2011 to 2020. In *S. sonnei*, the amoxicillin/clavulanic acid resistance rate was decreasing from 2014 to 2020, while the resistance of ampicillin and chloramphenicol decreased during the 2017–2020 period. However, resistance to ciprofloxacin and nalidixic acid increased considerably from 2017 to 2020. On the other hand, the rate of resistance to tetracycline, ampicillin and chloramphenicol in *S. flexneri* decreased moderately during 2017–2020, while ciprofloxacin resistance increased moderately (Figure 3). Specific details are shown in an additional file (Table S2).

Additionally, 96.5% of *S. sonnei* were MDR, presenting resistance to more than two classes of antibiotics, while 1.2% and 2.1% were resistant to two and one antibiotics, respectively. In *S. flexneri*, 74.4% were resistant to more than two antibiotics, while 22.6% and 7.8% were resistant to two and one antibiotics, respectively. In *S. boydii*, 64.9% were resistant to more than three antibiotics, while 24.4% and 9.2% were resistant to two and one antibiotics. An additional file shows this in more detail (Table S3).

## Discussion

Despite the efforts made to control bacterial diarrheal infections, *Shigella* is one of the leading causes of bacillary dysentery in Peru, Latin America, and other developing countries around the world.^1,4,5^ The age groups with the highest prevalence of shigellosis in these regions were children, especially those belonging to the age range from 0 to 5 years,^1,2^ which was reflected in this investigation (Table S1). Other age groups such as adults can also be affected by this disease, but the frequency rate in countries such as Peru is usually low, compared to the observed in children^18,28^; a situation different from that observed in developed countries such as the USA or England, which in recent years have reported an increase in shigellosis in adults; however, the way in which they acquire this infection is through sexual contact.^29,30^

One of the main limitations of this study is that most of *Shigella* strains were isolated in Lima (90.29%) (Figure 1). This is because many of the regions of Peru have limitations to carry out the diagnosis of *Shigella* by stool culture, often using traditional laboratory tests such as the presence of faecal leukocytes.^31^ For this reason, it is possible that the AMR rates presented in this analysis do not contemplate the real dimension of AMR at the level of the entire country. However, the number of strains included to achieve this study, as well as the period of time that it covers, is higher than those carried out by previous authors in Peru.^18,28^

Currently, several epidemiological studies have reported a change in the proportion of *Shigella* species around the world. In a study conducted in six Asian countries, *S. flexneri* was the most frequently isolated species in Bangladesh, China, Pakistan, Indonesia and Vietnam, while *S. sonnei* was the most predominant in Thailand.^6^ The changing epidemiology of shigellosis in developing countries located in Asia, Latin America and the Middle East shows a proportional decline of *S. flexneri*, with the simultaneous appearance of *S. sonnei.*^17^ In Peru, several studies have described *S. flexneri* as the most frequently isolated species, followed by *S. sonnei.*^18–20^ However, some changes have been observed in recent years. Guevara *et al*. were the first to describe the increased frequency of *S. sonnei*.^21^ On the other hand, Riveros and Ochoa found that 50% of the *Shigella* strains recovered since 2008 in the US Navy Medical Research Unit No. 06 (NAMRU-6) were identified as *S. sonnei*.^22^ In this study, a constant and significant increase in the frequency of *S. sonnei* (49.2%) in relation to *S. flexneri* (42.2%) was observed in a period of 10 years (Figure 2); a similar trend that has also been observed in previous studies.^12,32,33^ The increase in the isolation of *S. sonnei* was reported mainly in countries and regions that experienced rapid industrialization and sanitation development,^17^ as occurred in several Latin American countries where new economies emerged and growing industrialization contributed to improving water sanitation.^34^ In the last two decades, Colombia reports a constant increase of *S. sonnei* (52.7%) compared to *S. flexneri* (43.9%),^35^ while Brazil shows a marked predominance of the frequency of *S. sonnei* (88.2%) against *S. flexneri* (11.8%).^36^

The observed contradiction between the increase of *S. sonnei* versus better sanitation has not been determined, however, several hypotheses have been proposed.^17^ A possible explanation says *S. sonnei* and *Plesiomonas shigelloides* share a common O antigen, which may lead to natural cross-protective immunity in populations exposed to both microbial populations.^37^ These influences could have led to important changes in infectious disease patterns and epidemiological conditions, which could explain the observed change in the prevalence of *S. sonnei.*^38^ On the other hand, there is a possibility that the observed increase in *S. sonnei* is due to a greater capacity for speciation and clinical reporting.^38^ In addition, a greater survival and replication of *S. sonnei* inside *Acanthamoeba* has been reported, being able to act as a reservoir, which would favour a greater capacity to acquire AMR.^39^ As more countries increase their level of development and sanitation, *S. sonnei* could become a serious global public health problem.

On the other hand, the prevalence of *S. boydii* (7.9%) and *S. dysenteriae* (0.7%) were low compared to *S. sonnei* and *S. flexneri*. These findings agree with the low frequency of *S. boydii* reported in our country previously,^11^ however, it is frequently reported in Southeast Asia. Moreover, *S. dysenteriae* has not been reported in Peru for several years, except for the study conducted by Kosek *et al*., which in 2008 reported a frequency of 2.4% in the Peruvian Amazon.^19^  *S. dysenteriae* is most common in outbreak settings associated with civil unrest and refugee crises.^40,41^

Regarding the *Shigella* serotypes identified in this study, phase II was predominant in *S. sonnei*, which is consistent with some previous studies.^42^ On the other hand, eight different serotypes were observed in *S. flexneri* (1, 2, 3, 4, 5, 6, variants X and Y), being serotype 2a the most frequent (43.61%); this data coincides with two studies carried out in Peru, one by Koseck *et al*.^19^ and another by Guerrero *et al*.,^20^ reporting 33.1% and 48.15% of frequency corresponded to serotype 2a, respectively. Similar data were reported in Uruguay^43^ and Colombia,^44^ with 68% and 40% of cases, respectively.

Although *Shigella* serotypes share similar properties, they exhibit unique epidemiological characteristics. The true reason for the predominance of a serotype over a region is still not known with certainty.^45^ However, it is noted that some serotypes have a greater number of genetic virulence factors, such as *S. dysenteriae* serotype 1, which would influence their high mortality rate.^46^ Additionally, there is evidence that some of the serotypes are more likely to cause extraintestinal diseases, such as blood stream infections (*S. dysenteriae* serotype 1 and *S. flexneri* 3a),^47^ reactive arthritis (*S. flexneri* serotype 1, 2 and 2a, *S. sonnei* and *S. dysenteriae* type 1)^48^ and Hemolytic uremic syndrome (*S. dysenteriae* serotype 1).^49^ For all of these reasons, it is possible that the presence of specific *S. flexneri* or *S. sonnei* serotypes in our country increases the risk of causing an extraintestinal disease; however, this study only focuses on diarrheal manifestations.

The constant increase in AMR of *Shigella* species is a major problem in the treatment of *Shigella* gastroenteritis, especially the MDR strains.^50^ One of the main concerns surrounding *Shigella* is its ability to rapidly acquire antibiotic resistance, particularly *S. sonnei*, which can acquire resistance genes directly from *E. coli* through horizontal gene transfer. Several recent reports have suggested that *S. sonnei* is capable of sharing resistance plasmids through conjugation with commensal *E. coli.*^51,52^ In our study, approximately 84.2% of all *Shigella* strains showed MDR profiles, which is significantly higher than the rate of 41.6% (1762/4234) from the NARMS report (2005–2014).^53^ All MDR strains were highly resistant to traditional antimicrobials such as trimethoprim/sulfamethoxazole, tetracycline, ampicillin and chloramphenicol. This confirms that therapeutic alternatives for *Shigella* infections are scarcer, forcing the use of other groups or new antimicrobials. The reasons for the rapid accumulation of resistance are the excessive or inappropriate use of antibiotics in outpatients.^54,55^

*Shigella* resistance to trimethoprim/sulfamethoxazole, tetracycline, ampicillin and chloramphenicol has been reported by previous studies, particularly in *S. flexneri* and *S. sonnei*,^18^ a trend that continues until recent years, according to our analyses (Table 3). Something interesting is the decrease of AMR in *S. sonnei* to ampicillin and chloramphenicol during the years 2019–2020, with the consequent increase in resistance to fluoroquinolones (Figure 3). Since the acquisition of *Shigella* AMR to each of these antibiotic groups is attributed to different mechanisms, it is necessary to perform sequencing studies to understand the causes of such variation.^50^

Fluoroquinolones and third-generation cephalosporins are the alternative and first-line drugs recommended by the World Health Organization for the empirical treatment of shigellosis.^56^ However, in recent years, resistance to fluoroquinolones (ciprofloxacin and nalidixic acid) has increased significantly in all *Shigella* species, especially *S. sonnei* and *S. flexneri.*^13,38,50^ In this study, resistance to ciprofloxacin and nalidixic acid has increased considerably in *S. sonnei* since 2017, while the increase was moderate in *S. flexneri* and *S. boydii*. Regarding cephalosporins, resistance was less than 1.7% of the total strains analysed. However, our study shows that current resistance patterns have changed; several investigators suggest that empirical therapy should be modified according to these changes, and that treatment should be based on susceptibility patterns.^50^

Surveillance of AMR to azithromycin as a second-line antibiotic used to treat cases of shigellosis in Peru has been actively carried out since 2019. The lack of data corresponding to the years 2011–2018 did not allow a detailed analysis with this antibiotic, constituting the second limitation of our study. However, the data collected during 2020 (Table S4) suggest a high rate of resistance, especially against *S. sonnei* (65.1%) and *S. flexneri* (43.9%), a proportion comparable to that observed in other countries.^13,57^

### Conclusions

In conclusion, our findings show the predominance of *S. sonnei* in relation to *S. flexneri*, as well as a large proportion of resistance to trimethoprim/sulfamethoxazole, an antibiotic considered the first drug of choice to treat patients with inflammatory diarrhoea caused by *Shigella*. Also, an increasing trend in resistance to ciprofloxacin and nalidixic acid was observed. This increase to fluoroquinolones resistance is alarming and threatens the ability to control and manage this currently treatable disease. Finally, the implementation of an active *Shigella* surveillance is necessary in Peru and other developing countries to reinforce the prevention and control measures of shigellosis, Also, it is necessary to incorporate the use of molecular tools including Next Generation Sequencing for identification of novel mechanisms of AMR and the emergency of MDR *Shigella* strains.

## Supplementary Material

dlad110_Supplementary_Data

## Supplementary data

Tables S1 to S4 are available as Supplementary data at *JAC-AMR* Online.

## References

[1] GBD 2016 Diarrhoeal Disease Collaborators . Estimates of the global, regional, and national morbidity, mortality, and aetiologies of diarrhoea in 195 countries: a systematic analysis for the Global Burden of Disease Study 2016. Lancet Infect Dis 2018; 18: 1211–28. 10.1016/S1473-3099(18)30362-1 30243583 PMC6202444

[2] GBD 2016 Causes of Death Collaborators . Global, regional, and national age-sex specific mortality for 264 causes of death, 1980–2016: a systematic analysis for the Global Burden of Disease Study 2016. Lancet 2017; 390: 1151–210. 10.1016/S0140-6736(17)32152-9 28919116 PMC5605883

[3] GBD 2016 DALYs and HALE Collaborators . Global, regional, and national disability-adjusted life-years (DALYs) for 333 diseases and injuries and healthy life expectancy (HALE) for 195 countries and territories, 1990–2016: a systematic analysis for the Global Burden of Disease Study 2016. Lancet 2017; 390: 1260–344. 10.1016/S0140-6736(17)32130-X 28919118 PMC5605707

[4] GBD Diarrhoeal Diseases Collaborators . Estimates of global, regional, and national morbidity, mortality, and aetiologies of diarrhoeal diseases: a systematic analysis for the Global Burden of Disease Study 2015. Lancet Infect Dis 2017; 17: 909–48. 10.1016/S1473-3099(17)30276-1 28579426 PMC5589208

[5] Kotloff KL, Riddle MS, Platts-Mills JA et al Shigellosis. Lancet 2018; 391: 801–12. 10.1016/S0140-6736(17)33296-8 29254859

[6] Seidlein LV, Kim DR, Ali M et al A multicentre study of *Shigella* diarrhoea in six Asian countries: disease burden, clinical manifestations, and microbiology. PLoS Med 2006; 3: e353. 10.1371/journal.pmed.0030353 16968124 PMC1564174

[7] Strockbine NA, Bopp CA, Fields PI et al *Escherichia*, *Shigella*, and *Salmonella*. In: Jorgensen JH Carroll KC Funke G Pfaller MA Landry ML Richter SS and Warnock DW eds. Manual of Clinical Microbiology. ASM Press, 2015, 685–713.

[8] Coimbra RS, Lenormand P, Grimont F et al Molecular and phenotypic characterization of potentially new *Shigella dysenteriae* serotype. J Clin Microbiol 2001; 39: 618–21. 10.1128/JCM.39.2.618-621.2001 11158117 PMC87786

[9] Taneja N, Mewara A. Shigellosis: epidemiology in India. Indian J Med Res 2016; 143: 565–76. 10.4103/0971-5916.187104 27487999 PMC4989829

[10] WHO . Guidelines for the control of shigellosis, including epidemics due to *Shigella dysenteriae* type 1. 2005; https://iris.who.int/bitstream/handle/10665/43252/924159330X.pdf

[11] Puzari M, Sharma M, Chetia P. Emergence of antibiotic resistant *Shigella* species: a matter of concern. J Infect Public Health 2018; 11: 451–4. 10.1016/j.jiph.2017.09.025 29066021

[12] Gu B, Zhou M, Ke X et al Comparison of resistance to third-generation cephalosporins in *Shigella* between Europe-America and Asia-Africa from 1998 to 2012. Epidemiol Infect 2015; 143: 2687–99. 10.1017/S0950268814003446 25553947 PMC9151070

[13] Mahbubur R, Shoma S, Rashid H et al Increasing spectrum in antimicrobial resistance of *Shigella* isolates in Bangladesh: resistance to azithromycin and ceftriaxone and decreased susceptibility to ciprofloxacin. J Health Popul Nutr 2007; 25: 158–67. 17985817 PMC2753991

[14] Ranjbar R, Farahani A. *Shigella*: antibiotic-resistance mechanisms and new horizons for treatment. Infect Drug Resist 2019; 12: 3137–67. 10.2147/IDR.S219755 31632102 PMC6789722

[15] Tacconelli E, Carrara E, Savoldi A et al Discovery, research, and development of new antibiotics: the WHO priority list of antibiotic-resistant bacteria and tuberculosis. Lancet Infect Dis 2018; 18: 318–27. 10.1016/S1473-3099(17)30753-3 29276051

[16] Sirijatuphat R, Sripanidkulchai K, Boonyasiri A et al Implementation of global antimicrobial resistance surveillance system (GLASS) in patients with bacteremia. PLoS ONE 2018; 13: e0190132. 10.1371/journal.pone.0190132 29298323 PMC5752004

[17] Thompson CN, Duy PT, Baker S. The rising dominance of *Shigella sonnei*: an intercontinental shift in the etiology of bacillary dysentery. PLoS Negl Trop Dis 2015; 9: e0003708. 10.1371/journal.pntd.0003708 26068698 PMC4466244

[18] Baca C, Yupanqui L, Canales J et al Serotipos y susceptibilidad antimicrobiana de *Shigella* aisladas en un instituto de salud pediátrico de Lima, Perú entre enero y julio 2013. Rev Medica Hered 2014; 25: 73–9. 10.20453/rmh.v25i2.248

[19] Kosek M, Yori PP, Pan WK et al Epidemiology of highly endemic multiply antibiotic-resistant shigellosis in children in the Peruvian Amazon. Pediatrics 2008; 122: e541–9. 10.1542/peds.2008-0458 18710884 PMC6204332

[20] Barrantes CEG, Guillen A, Rojas LR et al Serotipos y resistencia antibiótica en *Shigella* spp aisladas de infecciones intestinales, Lima, 2012. Rev ECI Perú 2018; 10: 8.

[21] Guevara JM, Cipriani R, Giraldo D et al *Shigella sonnei*: ¿Está ocurriendo un cambio en nuestro medio? An Fac Med 2014; 75: 189–91. 10.15381/anales.v75i2.8390

[22] Riveros M, Ochoa T. Enteropatógenos de importancia en salud pública. Rev Peru Med Exp Salud Publica 2015; 32: 157–64. 10.17843/rpmesp.2015.321.1588 26102119

[23] Khalil IA, Troeger C, Blacker BF et al Morbidity and mortality due to *Shigella* and enterotoxigenic *Escherichia coli* diarrhoea: the Global Burden of Disease Study 1990–2016. Lancet Infect Dis 2018; 18: 1229–40. 10.1016/S1473-3099(18)30475-4 30266330 PMC6202441

[24] Magiorakos AP, Srinivasan A, Carey RB et al Multidrug-resistant, extensively drug-resistant and pandrug-resistant bacteria: an international expert proposal for interim standard definitions for acquired resistance. Clin Microbiol Infect 2012; 18: 268–81. 10.1111/j.1469-0691.2011.03570.x 21793988

[25] Bauer AW, Kirby WM, Sherris JC et al Antibiotic susceptibility testing by a standardized single disk method. Am J Clin Pathol 1966; 45: 493–6. 10.1093/ajcp/45.4_ts.493 5325707

[26] CLSI . Performance Standards for Antimicrobial Susceptibility Testing*—*Thirtieth Edition: M100. 2020.

[27] Jarlier V, Nicolas MH, Fournier G et al Extended broad-spectrum beta-lactamases conferring transferable resistance to newer beta-lactam agents in Enterobacteriaceae: hospital prevalence and susceptibility patterns. Rev Infect Dis 1988; 10: 867–78. 10.1093/clinids/10.4.867 3263690

[28] Lluque A, Mosquito S, Gomes C et al Virulence factors and mechanisms of antimicrobial resistance in *Shigella* strains from periurban areas of Lima (Peru). Int J Med Microbiol 2015; 305: 480–90. 10.1016/j.ijmm.2015.04.005 25998616 PMC4461498

[29] McCrickard LS, Crim SM, Kim S et al Disparities in severe shigellosis among adults—foodborne diseases active surveillance network, 2002–2014. BMC Public Health 2018; 18: 221. 10.1186/s12889-018-5115-4 29415691 PMC5803893

[30] Dallman TJ, Charles H, Prochazka M et al Emergence of novel strains of *Shigella flexneri* associated with sexual transmission in adult men in England, 2019–2020. J Med Microbiol 2021; 70: 001437. 10.1099/jmm.0.001437 34665107 PMC8604172

[31] Guillén A, Lucho J. Retos y problemas en el diagnóstico microbiológico en diarrea. Rev Peru Med Exp Salud Publica 2011; 28: 116–20. 10.1590/S1726-46342011000100018 21537779

[32] Anderson M, Sansonetti PJ, Marteyn BS. *Shigella* diversity and changing landscape: insights for the twenty-first century. Front Cell Infect Microbiol 2016; 6: 45. 10.3389/fcimb.2016.00045 27148494 PMC4835486

[33] Mao Y, Cui E, Bao C et al Changing trends and serotype distribution of *Shigella* species in Beijing from 1994 to 2010. Gut Pathog 2013; 5: 21. 10.1186/1757-4749-5-21 23919811 PMC3750644

[34] The World Bank/IBRD IDA . The world bank health data for Latin America. 2017; https://data.worldbank.org/topic/health?end=2012&locations=ZJ-Z4-Z7&start=1978

[35] Rodríguez EC, Bautista AM, Montaño LA et al Laboratory-based surveillance of *Shigella* spp. from human clinical cases in Colombia, 1997–2018. Biomédica 2021; 41: 65–78. 10.7705/biomedica.5113 33761190 PMC8055590

[36] Sousa MÂB, Mendes EN, Collares GB et al *Shigella* in Brazilian children with acute diarrhoea: prevalence, antimicrobial resistance and virulence genes. Mem Inst Oswaldo Cruz 2013; 108: 30–5. 10.1590/S0074-02762013000100005 23440111 PMC3974317

[37] Sack DA, Hoque S, Etheridge M et al Is protection against shigellosis induced by natural infection with *Plesiomonas shigelloides*? Lancet 1994; 343: 1413–5. 10.1016/S0140-6736(94)92531-3 7910890

[38] Sati HF, Bruinsma N, Galas M et al Characterizing *Shigella* species distribution and antimicrobial susceptibility to ciprofloxacin and nalidixic acid in Latin America between 2000–2015. PLoS ONE 2019; 14: e0220445. 10.1371/journal.pone.0220445 31374081 PMC6677304

[39] Saeed A, Johansson D, Sandström G et al Temperature depended role of *Shigella* flexneri invasion plasmid on the interaction with *Acanthamoeba castellanii*. Int J Microbiol 2012; 2012: 917031. 10.1155/2012/917031 22518151 PMC3299343

[40] Kernéis S, Guerin PJ, von Seidlein L et al A look back at an ongoing problem: *Shigella dysenteriae* type 1 epidemics in refugee settings in Central Africa (1993–1995). PLoS ONE 2009; 4: e4494. 10.1371/journal.pone.0004494 19214226 PMC2636862

[41] Octavia S, Lan R. Chapter 65—*Shigella* and shigellosis: genetics, epidemiology and pathogenesis. In: Tang YW Sussman M Liu D Poxton I and Schwartzman J eds. Molecular Medical Microbiology: Academic Press, 2015, 1147–68.

[42] Chung The H, Bodhidatta L, Pham DT et al Evolutionary histories and antimicrobial resistance in *Shigella flexneri* and *Shigella sonnei* in Southeast Asia. Commun Biol 2021; 4: 353. 10.1038/s42003-021-01905-9 33742111 PMC7979695

[43] Mota MI, Varela G, del Pilar Gadea M et al Serotipos, perfil plasmídico y antibiotipos de cepas de *Shigella flexneri* aisladas de niños menores de 5 años con diarrea sanguinolenta usuarios de los servicios de Salud Pública. Rev Médica Urug 2005; 21: 30–6.

[44] Bellorín I, Urbina G, González F et al Serotipos y resistencia antimicrobiana de cepas de *Shigella flexneri* aisladas de niños con diarrea aguda: relación entre el serotipo y la severidad del episodio. Rev Soc Venez Microbiol 2008; 28: 110–5.

[45] Muthuirulandi Sethuvel DP, Devanga Ragupathi NK, Anandan S et al Update on: *Shigella* new serogroups/serotypes and their antimicrobial resistance. Lett Appl Microbiol 2017; 64: 8–18. 10.1111/lam.12690 27783408

[46] Pakbin B, Brück WM, Brück TB. Molecular mechanisms of *Shigella* pathogenesis; recent advances. Int J Mol Sci 2023; 24: 2448. 10.3390/ijms24032448 36768771 PMC9917014

[47] Sharma S, Arora A. *Shigella flexneri* bacteremia in adult. J Lab Physicians 2012; 4: 65–6. 10.4103/0974-2727.98682 22923931 PMC3425274

[48] Mazumder RN, Salam MA, Ali M et al Reactive arthritis associated with *Shigella dysenteriae* type 1 infection. J Diarrhoeal Dis Res 1997; 15: 21–4. 9308297

[49] Banerjee S . Hemolytic uremic syndrome. Indian Pediatr 2009; 46: 1075–84. 20061586

[50] Wang Y, Ma Q, Hao R et al Antimicrobial resistance and genetic characterization of *Shigella* spp. in Shanxi Province, China, during 2006–2016. BMC Microbiol 2019; 19: 116. 10.1186/s12866-019-1495-6 31142259 PMC6542020

[51] Qu F, Ying Z, Zhang C et al Plasmid-encoding extended-spectrum β-lactamase CTX-M-55 in a clinical *Shigella sonnei* strain, China. Future Microbiol 2014; 9: 1143–50. 10.2217/fmb.14.53 25405884

[52] Rashid H, Rahman M. Possible transfer of plasmid mediated third generation cephalosporin resistance between *Escherichia coli and Shigella sonnei* in the human gut. Infect Genet Evol 2015; 30: 15–8. 10.1016/j.meegid.2014.11.023 25461693

[53] CDC . National antimicrobial resistance monitoring system for enteric bacteria (NARMS): human isolates surveillance report for 2014. 2016; https://www.cdc.gov/narms/pdf/2014-Annual-Report-narms-508c.pdf

[54] Pickering LK . Antimicrobial resistance among enteric pathogens. In: Finn A and Pollard AJ eds. Hot Topics in Infection and Immunity in Children IV. Springer, 2008, 154–63. 10.1007/978-0-387-73960-1_12 18193664

[55] Zhang W, Luo Y, Li J et al Wide dissemination of multidrug-resistant *Shigella* isolates in China. J Antimicrob Chemother 2011; 66: 2527–35. 10.1093/jac/dkr341 21859815

[56] Gendrel D, Cohen R. Diarrhées bactériennes et antibiotiques: les recommandations européennes. Arch Pédiatrie 2008; 15: S93–6. 10.1016/S0929-693X(08)74223-4 19000862

[57] Houpt ER, Ferdous T, Ara R et al Clinical outcomes of drug-resistant shigellosis treated with azithromycin in Bangladesh. Clin Infect Dis 2021; 72: 1793–8. 10.1093/cid/ciaa363 32239137

