# Supplementary material for: Trends in antimicrobial resistance of Shigella species in Peru, 2011–2020
Source: JAC Antimicrob Resist. 2023 Oct 26;5(5):dlad110. doi: 10.1093/jacamr/dlad110 (PMC10600570; doi:10.1093/jacamr/dlad110)
Supplement: dlad110_Supplementary_Data [file dlad110_supplementary_data.zip › Supplementary file 1.docx]

**Table S1.** Distribution of *Shigella* species by age and gender.

| **Age** | ***S. flexneri*** | ***S. sonnei*** | ***S. boydii*** | **Total*** |
| --- | --- | --- | --- | --- |
|  | **N= 704** | **N=821** | **N=131** | **N=1668** |
|  | **n= (%)** | **n= (%)** | **n= (%)** | **n= (%)** |
| **0-5** | 463 (66.77) | 569 (69.31) | 86 (65.65) | 1123 (67.33) |
| **6-11** | 98 (13.92) | 126 (15.35) | 24 (18.32) | 250 (14.99) |
| **12-18** | 20 (2.84) | 15 (1.83) | 6 (4.58) | 43 (2.58) |
| **19-26** | 11 (1.56) | 3 (0.37) | 0 (0) | 14 (0.84) |
| **27-59** | 6 (0.85) | 10 (1.22) | 2 (1.53) | 19 (1.14) |
| **60 ≥** | 6 (0.85) | 5 (0.61) | 1 (0.76) | 13 (0.78) |
| **ND** | 100 (14.2) | 93 (11.33) | 12 (9.16) | 206 (12.35) |
| **Gender** |  |  |  |  |
| **Male** | 305 (43.32) | 383 (46.65) | 59 (45.04) | 751 (45.02) |
| **Female** | 329 (46.73) | 377 (45.92) | 62 (47.33) | 775(46.46) |
| **ND** | 70 (9.94) | 61 (7.43) | 10 (7.63) | 142 (8.51) |

**ND:** No data recorded

*: The total includes *S. dysenteriae*

**Table S2.** Percentage of antibiotic resistance in *Shigella* strains from 2011 to 2020.

| **Species** | **Antibiotics** | **2011** | **2012** | **2013** | **2014** | **2015** | **2016** | **2017** | **2018** | **2019** | **2020** | **Total** |
| --- | --- | --- | --- | --- | --- | --- | --- | --- | --- | --- | --- | --- |
| ***S. sonnei*** |  | **N=96** | **N=85** | **N=81** | **N=80** | **N=56** | **N=124** | **N=40** | **N=96** | **N=80** | **N=83** | **N=821** |
|  | **SXT** | 95.8 | 95.3 | 98.8 | 97.5 | 98.2 | 97.6 | 100.0 | 89.6 | 100.0 | 96.4 | 96.6 |
|  | **TET** | 97.9 | 100.0 | 98.8 | 97.5 | 80.4 | 99.2 | 100.0 | 84.4 | 78.8 | 94.0 | 93.4 |
|  | **AMP** | 97.9 | 98.8 | 97.5 | 97.5 | 78.6 | 98.4 | 67.5 | 62.5 | 15.0 | 21.7 | 75.3 |
|  | **CHL** | 97.9 | 98.8 | 98.8 | 97.5 | 76.8 | 98.4 | 67.5 | 55.2 | 15.0 | 1.2 | 72.4 |
|  | **AMC** | 47.9 | 80.0 | 42.0 | 17.5 | 21.4 | 19.4 | 12.5 | 6.3 | 0.0 | 1.2 | 28.6 |
|  | **CIP** | 1.0 | 0.0 | 4.9 | 0.0 | 0.0 | 0.0 | 15.0 | 77.1 | 87.5 | 96.4 | 25.8 |
|  | **NA** | 1.0 | 0.0 | 1.2 | 1.3 | 0.0 | 4.0 | 37.5 | 38.5 | 88.8 | 97.6 | 25.6 |
|  | **NIT** | 1.0 | 2.4 | 1.2 | 0.0 | 0.0 | 0.8 | 0.0 | 0.0 | 0.0 | 1.2 | 0.7 |
|  | **CAZ** | 0.0 | 1.2 | 1.2 | 0.0 | 0.0 | 0.0 | 2.5 | 1.0 | 0.0 | 1.2 | 0.6 |
|  | **CTX** | 1.0 | 1.2 | 2.5 | 0.0 | 0.0 | 0.0 | 0.0 | 0.0 | 2.5 | 0.0 | 0.7 |
|  |  |  |  |  |  |  |  |  |  |  |  |  |
| ***S. flexneri*** |  | **N=81** | **N=115** | **N=86** | **N=37** | **N=43** | **N=87** | **N=98** | **N=64** | **N=36** | **N=57** | **N=704** |
|  | **SXT** | 82.7 | 85.2 | 90.7 | 78.4 | 93.0 | 86.2 | 85.7 | 78.1 | 91.7 | 66.7 | 84.1 |
|  | **TET** | 91.4 | 84.3 | 84.9 | 83.8 | 90.7 | 87.4 | 89.8 | 90.6 | 66.7 | 59.6 | 84.4 |
|  | **AMP** | 74.1 | 79.1 | 80.2 | 86.5 | 74.4 | 72.4 | 67.3 | 82.8 | 58.3 | 66.7 | 74.6 |
|  | **CHL** | 75.3 | 75.7 | 79.1 | 78.4 | 65.1 | 69.0 | 63.3 | 51.6 | 47.2 | 54.4 | 67.6 |
|  | **AMC** | 33.3 | 55.7 | 31.4 | 21.6 | 11.6 | 21.8 | 18.4 | 17.2 | 5.6 | 22.8 | 4.5 |
|  | **CIP** | 1.2 | 0.0 | 0.0 | 0.0 | 2.3 | 1.1 | 3.1 | 9.4 | 22.2 | 21.1 | 3.1 |
|  | **NA** | 2.5 | 0.0 | 3.5 | 0.0 | 4.7 | 2.3 | 6.1 | 4.7 | 8.3 | 1.8 | 27.6 |
|  | **NIT** | 1.2 | 0.0 | 1.2 | 0.0 | 0.0 | 1.1 | 1.0 | 0.0 | 0.0 | 1.8 | 0.7 |
|  | **CAZ** | 1.2 | 0.0 | 0.0 | 0.0 | 0.0 | 1.1 | 2.0 | 0.0 | 0.0 | 0.0 | 0.6 |
|  | **CTX** | 1.2 | 0.0 | 1.2 | 0.0 | 0.0 | 0.0 | 2.0 | 0.0 | 0.0 | 12.3 | 1.6 |
|  |  |  |  |  |  |  |  |  |  |  |  |  |
| ***S. boydii*** |  | **N=14** | **N=34** | **N=7** | **N=5** | **N=6** | **N=5** | **N=6** | **N=16** | **N=24** | **N=14** | **N=131** |
|  | **SXT** | 100.0 | 97.1 | 100.0 | 80.0 | 33.3 | 80.0 | 100.0 | 93.8 | 100.0 | 92.9 | 93.1 |
|  | **TET** | 92.9 | 82.4 | 100.0 | 20.0 | 66.7 | 100.0 | 100.0 | 81.3 | 87.5 | 85.7 | 84.0 |
|  | **AMP** | 42.9 | 50.0 | 71.4 | 20.0 | 66.7 | 40.0 | 83.3 | 87.5 | 83.3 | 78.6 | 64.9 |
|  | **CHL** | 21.4 | 2.9 | 14.3 | 20.0 | 0.0 | 20.0 | 0.0 | 0.0 | 12.5 | 0.0 | 7.6 |
|  | **AMC** | 14.3 | 17.6 | 0.0 | 0.0 | 0.0 | 0.0 | 16.7 | 12.5 | 4.2 | 14.3 | 8.4 |
|  | **CIP** | 0.0 | 0.0 | 0.0 | 0.0 | 0.0 | 0.0 | 0.0 | 6.3 | 25.0 | 28.6 | 9.2 |
|  | **NA** | 7.1 | 5.9 | 0.0 | 20.0 | 16.7 | 0.0 | 0.0 | 6.3 | 16.7 | 14.3 | 10.7 |
|  | **NIT** | 0.0 | 2.9 | 0.0 | 0.0 | 0.0 | 0.0 | 0.0 | 0.0 | 0.0 | 0.0 | 0.8 |
|  | **CAZ** | 7.1 | 0.0 | 0.0 | 0.0 | 0.0 | 0.0 | 0.0 | 0.0 | 0.0 | 0.0 | 0.8 |
|  | **CTX** | 0.0 | 0.0 | 0.0 | 0.0 | 0.0 | 0.0 | 0.0 | 0.0 | 0.0 | 7.1 | 0.8 |
| ***S. dysenteriae*** |  | **N=2** | **N=4** | **N=1** | **N=1** | **0** | **0** | **N=3** | **N=1** | **0** | **0** | **N=12** |
|  | **SXT** | 50.0 | 100.0 | 100.0 | 100.0 | 0.0 | 0.0 | 100.0 | 100.0 | 0.0 | 0.0 | 91.7 |
|  | **TET** | 0.0 | 25.0 | 100.0 | 0.0 | 0.0 | 0.0 | 66.7 | 0.0 | 0.0 | 0.0 | 33.3 |
|  | **AMP** | 0.0 | 50.0 | 100.0 | 0.0 | 0.0 | 0.0 | 66.7 | 100.0 | 0.0 | 0.0 | 50.0 |
|  | **CHL** | 0.0 | 25.0 | 100.0 | 0.0 | 0.0 | 0.0 | 0.0 | 0.0 | 0.0 | 0.0 | 16.7 |
|  | **AMC** | 0.0 | 25.0 | 0.0 | 0.0 | 0.0 | 0.0 | 0.0 | 0.0 | 0.0 | 0.0 | 8.3 |
|  | **CIP** | 0.0 | 0.0 | 0.0 | 0.0 | 0.0 | 0.0 | 0.0 | 100.0 | 0.0 | 0.0 | 16.7 |
|  | **NA** | 0.0 | 0.0 | 0.0 | 0.0 | 0.0 | 0.0 | 33.3 | 100.0 | 0.0 | 0.0 | 8.3 |
|  | **NIT** | 100.0 | 100.0 | 100.0 | 100.0 | 0.0 | 0.0 | 100.0 | 100.0 | 0.0 | 0.0 | 100.0 |
|  | **CAZ** | 0.0 | 0.0 | 0.0 | 0.0 | 0.0 | 0.0 | 0.0 | 0.0 | 0.0 | 0.0 | 0.0 |
|  | **CTX** | 0 | 0 | 0 | 0 | 0 | 0 | 0 | 0 | 0 | 0 | 0 |

**SXT:** trimethoprim/sulfamethoxazole, **TET:** Tetracycline, **AMP:** Ampicillin **CHL:** Chloramphenicol, **CIP:** Ciprofloxacin, **NA:** nalidixic acid, **AMC:** Amoxicillin/clavulanic acid, **NIT:** Nitrofurantoin, **CAZ:** ceftazidime, **CTX:** Cefotaxime.

**Table S4.** Antibiotic resistance to azithromycin in *Shigella* strains by subspecies in 2020.

| **Antibiotic** | ***S. sonnei***  **N=83**  **n (%)** | ***S. flexneri***  **N=57**  **n (%)** | ***S. boydii***  **N=14**  **n (%)** | **Total**  **N=154**  **n (%)** |
| --- | --- | --- | --- | --- |
| **AZM** | 54 (65.1) | 25 (43.9) | 5 (35.7) | 84 (54.5) |
